# Comorbidity of Asperger's syndrome and Bipolar disorder

**DOI:** 10.1186/1745-0179-4-26

**Published:** 2008-11-17

**Authors:** Michele Raja, Antonella Azzoni

**Affiliations:** 1 Servizio Psichiatrico di Diagnosi e Cura, Ospedale Santo Spirito in Sassia, Rome, Italy

## Abstract

**Background and objective:**

Asperger's Syndrome (AS) is a pervasive developmental disorder that is sometimes unrecognized, especially in the adult psychiatric setting. On the other hand, in patients with an AS diagnosis, comorbid psychiatric disorders may be unrecognized in the juvenile setting. The aim of the paper is to show and discuss some troublesome and complex problems of the management of patients with AS and comorbid Bipolar Disorder (BD).

**Methods:**

The paper describes three patients affected by AS and bipolar spectrum disorders.

**Results and conclusion:**

Mood stabilizers and 2^nd ^generation antipsychotics were effective in the treatment of these AS patients with comorbid BD, while the use of antidepressants was associated with worsening of the mood disorder.

It is of importance to recognize both the psychiatric diagnoses in order to arrange an exhaustive therapeutic program and to define specific and realistic goals of treatment.

## Introduction

Despite its increasing popularity as a distinct condition (included in the ICD-10 in 1993 and in the DSM-IV in 1994), the nosological status of Asperger's syndrome (AS) and its diagnostic validity remains uncertain. An astonishing 556% increase in pediatric prevalence of pervasive developmental disorders (PDD) has been reported between 1991 and 1997 [1]. This jump is probably due to heightened awareness and changing diagnostic criteria rather than to new environmental influences.

Both AS and autism persist into adulthood, but their phenotypic expression varies with age. AS may also be unrecognized in adulthood, although usually not forever. Some individuals with AS live almost normally and show good adaptation, while many can hardly cope and need supervision. Some cases are referred to psychiatric services for adults because of concurrent mental disorders or behavioral derangement, especially aggression and self-injury, rather than specific symptoms of AS. In these circumstances, the AS diagnosis is often overlooked. Since these cases appear odd and atypical in comparison with patients commonly observed in the adult psychiatric setting, they often receive several diagnoses in the course of time. The awareness of the AS diagnosis has been considered contingent on certain key professionals, who are interested in the area [2]. However, even when the correct diagnosis of AS or other PDD is made, it should not be considered necessarily exhaustive. It is of importance also to recognize comorbid psychiatric disorders, especially if successfully treatable.

Comorbid psychiatric conditions are frequent in patients with PDD. Patients with AS often present eccentricities, emotional lability, impairments in social functioning, anxiety and obsessive traits, demoralization, suicidal ideation, tempers, coldness, defiance, motor and phonic tics, repetitive behaviors, and stereotypies, that can mimic other mental illnesses [3]. The differential diagnosis with true comorbidity of schizophrenia, BD or anxiety disorders is not always easy. Children with PDD have a two-to-six-times greater risk of experiencing comorbid psychiatric conditions than their normal peers [4-6]. Awareness of the problem is increasing but available evidence on the topic is scanty. Psychiatric comorbidity of AS has been often cited but not well examined. There are very few systematic studies on psychiatric comorbidity in PDD [7-10], and only one in AS [11]. Clinicians treating children report a high comorbidity with Attention deficit hyperactivity disorder (ADHD), Oppositional defiant disorder, Depressive disorders, and Bipolar disorder [7].

Data on BD and AS comorbidity are inconsistent. McElroy [12] emphasizes that bipolarity is a marker for comorbidity, and comorbid disorders, especially multiple conditions occurring when a patient is young, may be a marker for bipolarity. However, most studies [7-9,11], evidence Unipolar depression as the most common mood disorder in patients with PDD, while only one report by Munesue et al [10] suggests that BD might be the most frequent. Several factors could account for this discrepancy.

First, as discussed by Frazier et al [13], it is difficult to ascertain the rate of comorbidity between AS and BD since the diagnosis of AS is currently used rather indiscriminately, referring to a heterogeneous group [14], and the actual incidence of pediatric BD is probably underestimated until the definition of bipolarity in children is more fully agreed upon. Second, BD often begins in childhood or early adolescence with the clinical features of unipolar depression, acute psychosis, or comorbid disorder (e.g., ADHD, obsessive-compulsive disorder (OCD), panic attack, or eating disorder), while manic symptoms appear later. As a consequence, the rate of bipolar diagnosis, can increase with the mean age of studied population. Third, the current classification of mood disorders has poor reliability and validity. According to DSM-IV-TR, the differential diagnosis between unipolar depression and BD II should be based on the lifetime presence of four days of hypomania. Information on mild symptoms overlapping with manifestations of well-being is subject to recall bias, unreliable evaluation, misinterpretation, incoherence. Furthermore, the source of information (patient, relatives, social institutions) can suggest different conclusions. Widening or narrowing the criteria for the definition of hypomania modifies substantially the ratio between unipolar and bipolar II depression [15].

Notwithstanding such gray area, growing evidence suggests that PDD and BD frequently co-occur. Unfortunately, most studies do not explicit the number of cases with AS since they predate DSM-IV [16-18]. In a clinical sample of 727 children, 52 met criteria for PDD, 114 met criteria for mania, and 14 of 52 children with PDD met criteria also for BD (2% of all referrals, 12% of children with BD, and 27% of children with PDD) [19]. In a consecutive series of adult patients referred with a diagnosis of autism spectrum disorder, 7% had BD [20]. Autism spectrum disorder, BD and Tourette syndrome were found to co-occur at a greater than chance expectation in the study of Kerbeshian & Burd [21].

Also family data suggest an etiological link between AS and BD. DeLong and Dwyer [22] found that relatives of probands with PDD had a 4.2% prevalence of BD and that the prevalence was highest among relatives of probands with AS (6.1% versus 3.3% for relatives of probands with autism). Gillberg & Gillberg [23] found that 4 (17%) of 23 patients with AS and 3 (13%) of 23 patients with autism had a family history of affective disorder. Comparing children affected by autistic spectrum disorders with and without identifiable neurological disorder that could account for their autism, DeLong and Nohria [24] found that the latter had a higher rate of family history of affective disorder. On the contrary, Piven et al [25] found that major depression, but not BD, had higher lifetime prevalence in the parents of autistic probands in comparison with the general population.

Interestingly, a family history of BD may influence the phenomenology of patients with PDD. In subjects with autism spectrum disorder and a family history of BD, many features of childhood BD have been observed, including affective extremes, cyclicity, obsessive traits, neuro-vegetative disturbances, special abilities, and regression after initial normal development. On the other hand, subjects with autism spectrum disorder and without a family history of BD showed less florid agitation, fearfulness, and aggression, and were of lower functioning [26].

In a previous series of patients, we described common clinical features of patients with AS in the emergency psychiatric setting and discussed the differential diagnosis with psychotic disorders [27]. Here, we present three subjects affected by AS and concomitant bipolar spectrum disorders that outline some clinical features of these patients and discuss some troublesome and complex problems of their management.

### Case 1

A 19-year-old girl was admitted to a psychiatric intensive care unit and committed for psychomotor agitation, suicidal ideation, violent behavior against her parents. Her father suffered from anxiety, had overvalued somatic concerns, and was treated with alprazolam. Her paternal grandfather had suffered from anxiety and depression, with hypochondriac ideation. A maternal uncle, with a bewildering temperament and suffering from periodic acute crises with paranoid ideas, had been admitted to psychiatric wards and had attempted suicide in the past.

Patient's delivery at term was normal. Mild delay in walking and precocious speech were reported. In her childhood, the patient was hyperactive, restless and had difficulties in relationships with her fellows. She was afraid of her contemporaries and preferred to spend her time with adults. When she was nine, she presented a suspected seizure during sleep. Cerebral MRI and EEG were normal. She was treated with carbamazepine 400 mg/day for 5 years. Until the junior high school, her school outcomes were good. She had an excellent memory and wrote poetries and novels. She did not like television programs or cartoons, except in a period of time during which she loved to see the same scenes of the film Cinderella, endlessly. At junior high school, she was anxious, nervous, agitated. Nocturnal enuresis appeared. The patient was visited by a psychiatrist who made the diagnosis of "psychosis" and treated her with haloperidol and paroxetine. When she was fifteen, obsessive preoccupations about sex first appeared. She was troubled by the fact that "men and women are different". She often caught far and indirect references to this difference in people's speech, television, news papers or books and became upset or agitated. She presented similar reactions when she grasped remote references to "the difference between North and South". She had gone by her self and had broken off any contact with her fellows. When she was seventeen, she left the school. A psychiatrist prescribed olanzapine (10 mg/day) with moderate improvement. The drug was withdrawn for severe weight gain and substituted with risperidone. However, this last drug seemed to be less effective. One year before admission, the patient was visited by a psychiatrist who made the diagnosis of bipolar disorder (BD) and borderline personality disorder and prescribed paroxetine 40 mg/day, ox-carbazepine 600 mg/day, haloperidol 2.5 mg/day, and alprazolam 2 mg/day. Ten days before admission, the patient withdrew haloperidol by her self. On visit, she was lucid, oriented, anxious, agitated, and poorly cooperating. Abnormal face and clumsiness were evident. Speech was scanty and poor of content. There were neither hallucinations nor typical delusions. However, bizarre ideas and obsessions were prominent. Currently, she had no hobby or interest, did not watch TV, read books or papers, hear music, fearing to run into love or sexual contents. She was afraid to have a bath because the imagine of her body was disturbing and insisted on evacuating only every other day. A mixed mood state was evident. Dysphoria, agitation, decreased need for sleep, talkativeness, hostility, and aggressiveness against objects and people were associated with depressed mood, hopelessness, sense of guilty, suicidal thoughts. Blunt affect and social retirement were also prominent. Brain MRI was normal. EEG showed diffuse 5–6 Hz rhythm. WAIS-R revealed an I.Q. of 70 (verbal: 88; performance: 54). She met the DSM-IV criteria for AS and BD, mixed state. The first diagnosis was made for the first time and was immediately accepted by her treating psychiatrist. As soon as her parents were instructed about the clinical features of AS, they recognized them in patient's history. Although they were informed that the core symptoms of AS are not responsive to treatment, they were relieved by the fact that symptoms and behavior of her daughter, until then considered unusual and strange, were typical manifestations of a described disorder. She was treated with ox-carbazepine 600 mg b.i.d. and risperidone 2 mg b.i.d., with moderate improvement of mood symptoms, behavioral disorder, and global functioning.

### Case 2

A 25-year-old man came to visit for behavioral disorder. In the previous years, he had been visited by several psychiatrists who had made different diagnoses including "infantile psychosis", mental retardation, OCD, and depression. Patient's delivery at term and psychomotor development were normal. There was no delay in language acquisition. At age 5, he was excessively introverted and impaired in behaviors to regulate social interaction and communication. However, he played with his contemporaries. At junior high school, difficulties in mathematics were prominent. At high school, outcomes were poor. He was not able to develop appropriate peer relationships and presented lack of social and emotional reciprocity. In the past, he had auditory hallucinations and persecutory delusions, when his beloved uncle was ill. After his grandmother's death, he presented an episode of major depression. He was treated with paroxetine (20 mg/day), with initial improvement, followed by hypomanic switch. He became hyperactive, and intrusive (he asked people personal embarrassing questions), and paroxetine was withdrawn.

On visit, motor clumsiness, abnormal face, inappropriate eye-to-eye gaze, unfit facial expression, and bizarre body postures and gestures were evident. Patient's parents criticized him because "he moved graceless". The patient had a severe dyscalculia but his memory was prodigious. He had a restricted and pervasive field of interest in films and music. He was able to remember the names of actors, set-designers, and costumer designers of the films he had seen. He was also remarkably able to imitate people. He had no friend and spent his time alone at home, watching movies in television or hearing music. Obsessions and compulsions were present. He was compelled to touch objects many times, to repeat phrases and to count up to one hundred, continuously. Currently, he had some reference and persecutory ideas, but no delusions. In the last years, episodes of aggressiveness and violence against his father were reported. His father tended to criticize the patient considering him listless and lazy, rather than ill. The patient met the criteria of AS. His EEG and brain MRI were normal. In the past, he had suffered from an episode of major depression and antidepressants-induced hypomania pointing to a lifetime comorbid diagnosis of bipolar spectrum diagnosis. Retrospectively, it was not easy to ascertain whether the periodical recrudescence of patient's behavioral disorders were related to exacerbations of his mood disorder or not. Although mood symptoms were currently under control, he continued to have difficulty in several areas. The description of AS clinical features to patient and his relatives had a benefic impact. For the first time, his father realized that patient's most odd and troublesome behaviors were symptoms of a mental illness and not expression of naughtiness, indolence, or neglectfulness.

### Case 3

A thirty-year-old man was referred to psychiatric visit. None of his relatives suffered from psychiatric disorder but a sister of his maternal grandmother who withdrew any social relationship at age of thirty, and decided to see only her mother and her sister until death.

He presented infantile face, feminine timbre of voice, and motor clumsiness. Patient's delivery at term was uneventful. During the first year of life, he suffered from rhinitis with respiratory distress. Since then, his parents noted he was nervous. A few months later, facial tics appeared, worsened by emotions. At age three, dysarthria and stuttering appeared. Primary enuresis was present until age of six. At primary school, dyslexia (involving both letters and sentences, numbers and musical notes), and dysgraphia were noted. He presented better cognitive function in mathematics than in humanistic matters. He was impaired in non-verbal behaviors regulating social interaction, was not able to develop peer relationships appropriate to developmental level, and presented lack of social and emotional reciprocity. In childhood, he developed special interest in electric circuits, electric devices, bulbs, and in computers, acquiring a special ability. In his twenties, he developed a dominating interest for car races, spending most time watching them on TV or discussing them during countless psychotherapeutic sessions. He attended the faculty of engineering, passing a few exams, with low grades. Only rarely, he passed exams with high grades. In these occasions, his professors noted he had found the solution to problems in an original way, different from that suggested during the lessons. At age of thirty, he left the university and found a sheltered job as an expert of computers. He had never either associated with contemporaries or had friends. In the past, several periods of depressed mood, psychomotor inhibition, loss of energy, diminished interests, and poor functioning had occurred, during which he had been treated with antidepressants. These drugs had caused agitation, insomnia, flight of ideas, and distractibility and had been withdrawn.

He was nice, had a great sense of humor, and was exceptionally able to make people laugh and to imitate people. He stayed always alone, had no interest typical of his age and seldom left home. He had the compulsion to wear only shirts and pullovers with short neck, had sexual obsessions and compulsions, wishing and fearing to assault women wearing a skirt, and obsessions of committing suicide. He was often anxious and depressed and sometimes impulsively aggressive against people or violent against objects. He had never had delusions but only bizarre, esoteric ideas. Episodically, he had olfactory, visual, and auditory hallucinations, and feelings of depersonalization and derealization. He was also affected by adrenal insufficiency and hypothyroidism and therefore assumed corticosteroids, mineral corticoids, and thyroxin. During periods of increased doses of corticosteroids, his mood worsened with dysphoric excitement and suicidal thoughts. WAIS-R revealed an I.Q. of 108 (verbal: 105; performance: 110). He was treated with risperidone (1,5 mg/day) and ox-carbazepine (1200 mg/day), with marked improvement. Hallucinations vanished, his mood improved and became stable, obsessive sexual and suicidal thoughts lessened. Although his global functioning significantly improved, social and emotional interaction, peer relationships, interests and activities remained scanty.

## Discussion

Since 1994, when we recognized the first case of AS and became familiar with this syndrome [28], we diagnosed AS in 14 adult psychiatric patients, in the hospital and in the outpatient setting. Three of them (21.4%), described here, were also affected by bipolar spectrum disorder. In two of them, bipolarity appeared clearly only during antidepressant treatment. Besides DSM-IV criteria of AS, the patients presented common features reported in patients affected by PDD, including obsessive-compulsive symptoms, physical abnormalities, motor clumsiness, aggressive or violent behavior especially against relatives, sense of humor or ability to imitate, and history of different psychiatric diagnoses.

Usually, the diagnosis of AS has a great impact. Despite the absence of a cure for AS, the awareness of its distinctive clinical features can be helpful and important for rehabilitation and differentiate response to treatment and comorbid conditions. Almost invariably, patients and relatives are relieved by the diagnosis of a biologically based disorder that accounts for symptoms and behaviors perceived (sometimes also by treating clinicians) as mysterious, unusual, and frustrating.

Our patients had received numerous diagnoses in the past, but none of them had received the diagnosis of PDD. All of them had been visited only by adult psychiatrists but the first case, visited by a child neuro-psychiatrist who had focused his assessment on a suspected seizure, however. Although the prescribed psychotropic drug treatments clearly suggest that the treating psychiatrists had recognized the depressive and the manic symptoms, only the first patient had received a diagnosis of BD. Probably, the concurrent unusual and overwhelming psychiatric symptoms unrelated to BD had induced clinicians to delay a specific psychiatric diagnosis. In the first case, the psychiatrist added a generic diagnosis of personality disorder to the BD diagnosis, perhaps to account for the concurrent atypical symptoms. None of the past diagnoses had captured patients' symptoms completely.

### Implications for therapy

As usual in the case of patients who continue to present major psychiatric symptoms despite treatment, the described patients had been prescribed many psychotropic drugs in the course of years. In general, treatment resulted in remission of BD symptoms and improvement of behavior and global functioning, although a sense of dissatisfaction accompanied the management of the patients.

The literature on the psychopharmacological treatment of AS is essentially constituted by case reports or small cases series. The results are poorly consistent. A pre-requisite to assess the effectiveness of treatment is the correct evaluation of comorbid psychiatric disorders. Since AS is associated with long-term disability and there are no specific pharmacological treatments for the core deficits of the disorder, unrecognizing the comorbid BD may be associated with omitting effective treatment. On the other hand, unrecognizing AS may induce confusion in the evaluation of treatment and arrangement of rehabilitation strategy. The effectiveness of treatment may be overlooked, with the risk of inappropriate drug switch or withdrawal. Another possibility is the fallacious conclusion that antidepressants, mood stabilizers and antipsychotics of 2nd generation "are partially effective in AS".

The first step in treating any type of complicated BD is mood stabilization. In most cases, a significant improvement of comorbid disorder is usually seen. In accordance with this general rule, effectiveness of valproate [29] or lithium [30,31] has been reported in the treatment of patients with PDD and affective symptoms. Factors that may suggest a positive response to mood stabilizing agents include a family history of BD, hyperactivity, a definite cyclic component of symptomatic behaviors, sustained laughter, irritability, not stereotypic giddiness, or the presence of many symptom criteria for BD [30]. In the presented series, a favorable response to mood stabilizers and 2^nd ^generation antipsychotics was observed in cases 1 and 3.

A problematic topic is the treatment of obsessive-compulsive symptoms that are frequent both in patients with AS [27] and BD [32,33]. In the case of AS-BD comorbidity, it may be difficult to attribute obsessive-compulsive symptoms to one of the two syndromes. As a consequence, treatment strategy is not easy. Selective serotonin reuptake inhibitors (SSRI) are effective in the treatment of OCD. However, in the case of OCD-BD comorbidity, mood stabilizers or 2^nd ^generation antipsychotics are generally considered first choice-treatment [34-36]. As in other conditions comorbid with BD, the use of antidepressants can worsen the clinical condition (cases 2 and 3). Antidepressants, including SSRI, have been shown to induce mania in some patients with AS [37] and also to worsen aggressive behavior. Before using SSRI in the treatment of depressive or obsessive-compulsive symptoms of AS, the comorbid presence of BD or bipolar familiarity should be excluded.

## Abbreviations

ADHD: Attention Deficit Hyperactivity Disorder; AS: Asperger Syndrome; BD: Bipolar Disorder; OCD: Obsessive-Compulsive Disorder; PDD: Pervasive Developmental Disorders; SSRI: Selective Serotonin Reuptake Inhibitors

## Authors' contributions

MR and AA visited the cases, contributed to the study design and to the discussion. Both authors read and approved the final manuscript.

## Consent

Informed consent was obtained from the patients for publication of this case report.
